# Exploring 3D Printing in Drug Development: Assessing the Potential of Advanced Melt Drop Deposition Technology for Solubility Enhancement by Creation of Amorphous Solid Dispersions

**DOI:** 10.3390/pharmaceutics16121501

**Published:** 2024-11-22

**Authors:** Nabil Lamrabet, Florian Hess, Philip Leidig, Andreas Marx, Thomas Kipping

**Affiliations:** 1 Merck Life Science KGaA, Frankfurter Straße 250, 64293 Darmstadt, Germany; 2 Department of Biopharmaceutic and Pharmaceutical Technology, Institute of Pharmacy, University of Greifswald, Felix-Hausdorff-Straße 3, 17487 Greifswald, Germany

**Keywords:** 3D printing, advanced manufacturing, Advanced Melt Drop Deposition, amorphous solid dispersions, solubility enhancement

## Abstract

**Background:** Melt-based 3D printing technologies are currently extensively evaluated for research purposes as well as for industrial applications. Classical approaches often require intermediates, which can pose a risk to stability and add additional complexity to the process. The Advanced Melt Drop Deposition (AMDD) technology, is a 3D printing process that combines the principles of melt extrusion with pressure-driven ejection, similar to injection molding. This method offers several advantages over traditional melt-based 3D printing techniques, making it particularly suitable for pharmaceutical applications. **Objectives:** This study evaluates the AMDD printing system for producing solid oral dosage forms, with a primary focus on the thermo-stable polymer polyvinyl alcohol (PVA). The suitability of AMDD technology for creating amorphous solid dispersions (ASDs) is also examined. Finally, the study aims to define the material requirements and limitations of the raw materials used in the process. **Methods:** The active pharmaceutical ingredients (APIs) indometacin and ketoconazole were used, with PVA 4-88 serving as the carrier polymer. Powders, wet granulates, and pellets were investigated as raw materials and characterized. Dissolution testing and content analyses were performed on the printed dosage forms. Solid-state characterization was conducted using differential scanning calorimetry (DSC) and X-ray diffraction (XRD). Degradation due to thermal and mechanical stress was analyzed using nuclear magnetic resonance spectroscopy (NMR). **Results/Conclusions:** The results demonstrate that the AMDD 3D printing process is well-suited for producing solid dosage forms. Tablets were successfully printed, meeting mass uniformity standards. Adjusting the infill volume from 30% to 100% effectively controlled the drug release rate of the tablets. Solid-state analysis revealed that the AMDD process can produce amorphous solid dispersions with enhanced solubility compared to their crystalline form. The experiments also demonstrated that powders with a particle size of approximately 200 µm can be directly processed using AMDD technology.

## 1. Introduction

Recent decades have witnessed a transformative shift in drug research. A deeper understanding of the evolution and progression of various diseases has enabled the creation of more targeted therapies [1]. This shift is evident in the emergence of personalized medicine, a paradigm that is reshaping both medicine and drug development [2]. A significant percentage of adverse drug effects, estimated between 75% and 85%, are attributed to inappropriate dosing or dose combinations [3,4]. Consequently, there is a growing demand for methods to customize dosage forms to meet the specific needs of individual patients [5]. However, current conventional pharmaceutical manufacturing processes fall short in this regard, as they do not readily allow for tailored dosing [6]. This challenge is where Additive Manufacturing, or 3D Printing (3DP), comes into play. 3DP offers significant advantages, such as the ability to fabricate complex solid oral dosage forms (SODFs), enabling the customization and personalization of medicines with individually tailored doses [7]. Moreover, 3DP allows the production of 3D structures with high shape complexity [8], which enables printing SODFs with modified drug release rates [9]. Additionally, 3DP can accelerate the drug development timeline from initial human clinical trials to mainstream medical care [10].

In the pharmaceutical industry, 3DP is revolutionizing traditional manufacturing processes, especially for SODFs [11]. This revolution is particularly important considering that a large proportion of new active pharmaceutical ingredients (API), around 70%, have poor solubility [12]. Enhancing solubility often involves producing amorphous solid dispersions (ASD). One method to produce ASD is through hot melt extrusion. During this process, the crystalline structure of the API is disrupted by both thermal and mechanical energy, and the amorphous components are embedded into a polymer matrix [13]. The traditional method for processing these ASDs into SODFs involves milling the extrudate and tableting it with the addition of excipients [14]. However, this process can be challenging in early clinical trials due to the need for various dosages and different tablet formulations, especially when only small quantities of API are available [15].

Fused deposition modeling (FDM) is gaining popularity in pharmaceutical research due to its affordability and compatibility with HME, a well-established technology in the pharmaceutical field [16]. FDM is an extrusion-based system that uses a filament as the starting material. This filament, produced through HME and capable of being drug-loaded [17], is fused by a heated printer extrusion head. The head moves along the x and y axes, depositing the material through a nozzle onto a building surface [18]. The quality of the final printed form depends on various parameters, including infill density, extrusion speed, layer height, and nozzle/building platform temperature [17,19,20]. Infill density is particularly important in personalizing medicine as it allows modification of drug release rates in SODF [21]. However, producing filaments for FDM presents challenges. They must have mechanical stability to be receptive to the print head, necessitating specific mechanical properties [22,23]. Consequently, a thorough evaluation of active ingredient-containing filaments is essential, as mechanical stability varies with each active ingredient, leading to time-consuming and resource-intensive investigations. Furthermore, while several thermoplastic polymers have been assessed for pharmaceutical applications, the number of pharmaceutically approved polymers is limited, and many lack the required properties for FDM [18,24].

A novel solution to these challenges is Advanced Melt Drop Deposition (AMDD) technology, as demonstrated in the Arburg Plastic Freeforming (APF) process [25]. AMDD stands out because it uses standard granulation for printing, a significant advantage over other melt-based techniques like FDM. Utilizing powder granulation allows production of SODFs without prior processing. Moreover, AMDD technology could further process pelletized extrudate using bulk material instead of filament strands, which eliminates the need for specific mechanical properties and enables on-demand sales.

**Figure 1:**
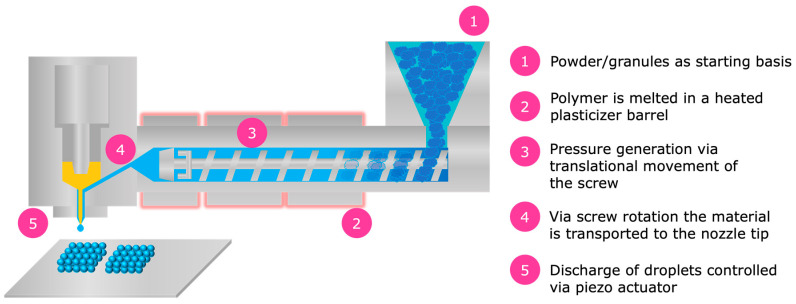
Illustration of the AMDD technology. 1. The API and polymer are introduced into the plasticizer barrel. 2. In the heated plasticizer barrel, the polymer–API mixture is melted. 3. A screw mechanism conveys the molten material toward the printing head, generating pressure through its motion. 4. The molten polymer–API mass is directed into the nozzle chamber. 5. The molten polymer–API mixture is then precisely ejected in the form of individual droplets, controlled by a piezo actuator that modulates a nozzle closure mechanism.

The printer in AMDD combines the principles of hot melt extrusion with pressurized ejection, like injection molding processes (Figure 1). In this system, the polymer granulate is heated and melted within a plasticizer barrel. A rotating screw then moves the molten material to the nozzle tip, generating high pressures (up to 600 bar) inside the barrel. This molten substance is then precisely discharged as individual droplets, controlled by a piezo actuator that modulates a nozzle closure mechanism. The high velocity of these droplets ensures their cohesion, enabling the construction of complex structures.

The aim of this study is to evaluate the AMDD technology for use in pharmaceutical applications. Initial applications of the AMDD system were described by Welsh et al., who printed a Dapivirine-containing vaginal ring from polyurethane and optimized its release rate. Furthermore, they significantly reduced the amount of active pharmaceutical ingredient (API) compared to conventional thermoplastic production techniques [26]. A second paper by Zhang et al. explored the AMDD process for the production of oral dosage forms. They studied Paracetamol, a highly soluble BCS (Biopharmaceutics Classification System) Class 1 drug, in a polymer matrix composed of hypromellose acetate succinate and a portion of polyethylene oxide. Their research focused on the impact of porosity on the release kinetics of swellable and erodible solid dosage forms [27].

This study sheds light on two areas that have not yet been explored in a pharmaceutical context: firstly, the impact of various initial intermediates on the processability. For this purpose, various intermediates, such as powder mixtures, wet granulates, and HME pellets, were examined. In this context, the limits of the system in terms of the processability of the starting materials are evaluated. The second focus is on the solubility enhancement of poorly soluble drugs through the formation of ASDs using the AMDD system. Ketoconazole (KTZ) and indometacin (IND), both BCS Class 2 drugs, were chosen as model compounds, covering both slightly acidic (IND) and slightly basic (KTZ) model substances. These were printed into a PVA matrix. The influence of the starting intermediates on the solid-state status of the ASDs was also examined. The analysis included particle size distribution (PSD), flow properties, rheology, differential scanning calorimetry (DSC), powder X-ray diffractometry (PXRD), tablet properties such as mass and scanning electron microscopy (SEM) images, determination of drug content with nuclear magnetic resonance (NMR), and high-performance liquid chromatography (HPLC), as well as in vitro dissolution studies.

## 2. Materials and Methods

### 2.1. Materials

Polyvinyl alcohol (Parteck^®^ MXP 4-88) as well as PVA 4-88 Flakes were purchased from Merck Life Science KGaA (Darmstadt, Germany). Indometacin (IND) was purchased from Sigma Aldrich (St. Louis, MO, USA). Ketoconazole (KTZ) was purchased from Fagron (Rotterdam, The Netherlands). All other reagents used for high-performance liquid chromatography (HPLC), and dissolution were of chromatography or analytical grade.

### 2.2. Methods

#### 2.2.1. Preparation of PVA Fractions

Individual fractions were produced from the PVA 4-88 flakes by cryo-milling with liquid nitrogen. For this, the Ultra-Centrifugal mill ZM-200 with a 12-tooth rotor (Retsch GmbH, Haan, Germany) was used. The milling was carried out at 18000 rpm using various sieves with a mesh size of 2000 µm, 1000 µm, 750 µm, and 500 µm.

#### 2.2.2. Physical Mixtures

Two physical mixtures were produced. The first physical mixture contained PVA and 10% KTZ and the second contained PVA and 10% IND, which were weighed and then mixed in a Turbula Mixer (Willy A. Bachofen AG, Muttenz, Switzerland) for 5 min at 47 rpm.

#### 2.2.3. Particle Size Distribution

The particle size distribution of the powder fractions was measured using a Malvern Mastersizer 2000^®^ laser diffractometer using a dry sampling system (Scirocco, 2000, Malvern, UK).

#### 2.2.4. Flow Properties

In accordance with the European Pharmacopoeia (Ph. Eur. 11.5) method 3 in Section 2.9.34, the bulk density of the milled PVA fractions was determined. For each fraction, five determinations were conducted. Initially, a graduated cylinder was filled with the test sample to a known volume. The mass of the powder was then accurately weighed, and the bulk density was calculated by dividing the mass by the volume.

Tapped density was determined according to Ph. Eur.2.9.34 method 1. The sample was introduced into a graduated cylinder and mechanically tapped until a constant volume was achieved. The mass of the powder was measured, and the tapped density was calculated as mass divided by the tapped volume. This procedure was repeated twice. The tapped density was determined with a tap volumeter STAV 2003 (J. Engelsmann AG, Ludwigshafen, Germany).

The angle of repose was determined according to Ph. Eur.2.9.36. The angle of repose was measured by allowing the powder to flow through a funnel onto a flat surface and forming a cone. The height and radius of the cone were measured, and the angle of repose was calculated using the tangent of the angle formed.

Carr’s Index was calculated as the percentage difference between the tapped density and bulk density, divided by the tapped density, multiplied by 100. It provided an insight into the flowability and compressibility of the powder.

Hausner Ratio was determined by dividing the tapped density by the bulk density. A higher ratio indicated poorer flow properties of the powder.

#### 2.2.5. Rheology

For the rheological analysis of the physical, a Haake Mars 60 plate–plate rheometer from (Thermo Fisher Scientific, Waltham, MA, USA) was utilized. The rheometer was equipped with aluminum single-use plates with a diameter of 25 mm. An amount of approximately 550 mg of the physical mixtures was applied to the lower plate and then heated to 210 °C. The top plate was then dropped onto the sample, setting a gap height of 1 mm. Any excess material that oozed out was trimmed off once the desired gap height was established. The temperature of 210 °C was maintained for 5 min before the samples were cooled to 180 °C. The viscosity and the phase angle δ were determined in the range of 210 °C to 180 °C. The cooling rate was 2 °C/min The upper plate moved, oscillating with a constant strain rate (0.1%) and frequency (6.28 rad/s). Each test was performed three times. Data analysis was performed using the HAAKE RheoWin software (Version 4.87.0006, Thermo Fisher Scientific, Waltham, MA, USA).

#### 2.2.6. Hot Melt Extrusion

The hot-melt extrusion process was conducted using a Pharma 11 extruder (Thermo Fisher Scientific, Waltham, MA, USA), equipped with a 2 mm die and a length of 3 cm. The screw configuration incorporated three mixing elements, ensuring thorough mixing of all ingredients. Further details on the screw configuration can be found in the Supplementary Materials (Figure S1). Material feeding was accomplished with a gravimetric feeder (Congrav^®^ OP 1 T, Brabender Technologie GmbH & Co. KG, Duisburg, Germany), and the screw speed was maintained at 300 rpm. All polymer/API binary mixtures as well as pure PVA were extruded with the specific settings outlined in Table 1. Subsequently, the extrudates were pelletized with the Brabender Pelletizer (Brabender GmbH & Co. KG, Duisburg, Germany) to achieve a consistent pellet size of 2 mm.

#### 2.2.7. Wet Granulation

For each of the two physical mixtures and the pure PVA, a wet granulation process was employed. This involved using deionized water as the granulating agent. Each physical mixture was individually introduced into a high-shear granulator BM30 (PEFRA & SC-GASTRO GmbH, Düsseldorf, Germany), operating at pace level 4. Throughout this procedure, 600 mL of deionized water was steadily applied to the mixture using a peristaltic pump PD5201 (Heidolph Instruments GmbH & CO. KG, Schwabach, Germany), which delivered the water through a 2 mm inner diameter silicone tube. The granulation process for each mixture was conducted over a duration of 45 min, including a 5 min post-mixing phase. Following the granulation, the granules from each batch were subjected to vacuum drying. This was performed in a vacuum drying cabinet VT6130M (Thermo Electron Corporation, Langenselbold, Germany) for 15 h at 80 °C and 200 mbar. Post-drying, the dried granules from each batch were then sieved using a mesh with a size of 2 mm to ensure uniformity in particle size.

#### 2.2.8. Computer-Aided Design

The geometry of the 3D Tablets was designed by the computer-aided design (CAD) software Fusion 360 (Autodesk, San Rafael, CA, USA). The 3D models were exported as a Standard Tessellation Language file format and were uploaded into the slicer software (Freeformer software v2.2 Arburg, Loßburg, Germany) of the 3D printer. In this present study, the standard geometry for each 3DP tablet (3DP) was 10 mm in diameter and 4 mm in height. The tablet design was modified inside the slicing software regarding infill volume from 30% to 100% infill.

#### 2.2.9. AMDD Printing

Prior to printing, physical mixtures underwent qualification on the Freeformer^®^ 200-3XArburg, Loßburg, Germany) following the manufacturer’s guidelines. This involved adjusting processing parameters like temperature and form factor (FF) (the width/height ratio of the droplet) to ensure correct droplet geometry and transparency. The FF, crucial for controlling droplet spacing and chain distance, affects the porosity and fill of the components [28]. Each new material requires a visual assessment of the surface to determine the appropriate FF for the Freeformer. To verify the settings for the FF, a series of droplets were printed in a strand formation. The dimensions of these droplets, specifically their width and height, were meticulously measured to accurately calculate the FF. Following this, a series of five cubic test pieces, each measuring 20 × 20 × 4 mm and incorporating different FFs, including the one derived from the measurements, were printed. Starting with the preliminary FF in the middle, each FF of the other cubes was changed with a step size of 0.02. The visual investigation of the printed strands and cubes was scrutinized under a Stemi 2000-C microscope (Carl Zeiss Microscopy GmbH, Jena, Germany) for a detailed evaluation. The goal was to produce a homogeneous surface on the cube while preventing shrinkage and warping. Table 2 shows the printing parameters for the placebo, KTZ-, and IND-loaded tablets. The printing parameters were adapted to the individual requirements of the API-PVA mixture. The final settings were determined after a visual evaluation of the printing results.

#### 2.2.10. Tablet Characterization

The 3DP tablets were measured in terms of their masses with an analytical balance from Mettler Toledo, type XP 105 Delta Range (Greifensee, Switzerland). For each infill volume 10 3DP tablets, produced from the different intermediates, were analyzed in order to determine the mass distribution according to the standard deviation criteria of the Ph. Eur. 2.9.5.

#### 2.2.11. Scanning Electron Microscope (SEM)

SEM pictures were executed using a MIRA3 TESCAN LMU microscope (TESCAN ORSAY HOLDING a.s, Brno, Czech Republic). The pictures were taken under a high vacuum with a wolfram cathode and an accelerating voltage of 5.0 kV. The magnification was from 23× to 484×. The tablets were sputtered with carbon.

#### 2.2.12. Differential Scanning Calorimetry (SEM)

The thermal properties of all raw materials, intermediates, and 3DP tablets were recorded on a DSC 3+ differential scanning calorimeter (Mettler Toledo, Gießen, Germany). The cell was purged with Nitrogen at a flow rate of 50 mL/min for all measurements. The sample quantity was between 5–7 mg and was weighed inside 40 µL aluminum pans. Prior to analysis, the aluminum pans were pierced by the autosampler. Each sample was subjected to measurements at two distinct heating rates, specifically at 5 °C/min and 30 °C/min, with the temperature range extending from −25 °C to 230 °C for both rates. The STARe SW 16.00 software (Mettler Toledo, Gießen Germany) was used to analyze the results.

#### 2.2.13. Powder X-Ray Diffraction (PXRD)

PXRD patterns were investigated using a Miniflex 300/600 from Rigaku Corporation (Tokyo, Japan) equipped with a CuKα anode. The 3DP tablets and the HME pellets were milled using the IKA Tube Mill 100 (IKA Werke GmbH & CO. KG, Staufen, Germany) to create the powder samples. The powder samples were measured with a continuous pace of 10 °/min over an angle range of 3° to 60° at a step size of 0.02°. The scanning was performed at an acceleration voltage of 40 kV and a current of 15 mA. The samples were measured once. The Analysis of results was conducted with the software PDXL 2 (Version 2.8.4.0, Rigaku Corporation, Tokyo, Japan).

#### 2.2.14. NMR Measurements

All 1H NMR spectra were recorded at 25 °C on a 700 MHz Bruker Avance III (Bruker Corporation, Billerica, MA, USA) spectrometer equipped with a cryocooled TCI probe. 1H NMR was recorded with 30° excitation. The FID was digitized with 64k data points, zero-filled to 128k points, and multiplied with an exponential function (lb 0.3) prior to Fourier transformation. The relaxation delay was set to 10s for quantitative measurements. The content of API within a formulation was determined with certified maleic acid as the quantification standard. Therefore, about 10 mg of reference standard and the sample was exactly weighted and dissolved in DMSO-d6.

#### 2.2.15. Content

All intermediates and 3DP tablets were dissolved to determine the content uniformity. The tablets as well as the HME pellets were milled at 25.000 rpm for 25 s using an IKA tube mill 100 (IKA-Werke GmbH Co. KG, Staufen, Germany). All samples were dissolved in a 100 mL volumetric flask and filtered through a 0.45 µm membrane filter. The dissolved samples (n = 2) were analyzed using HPLC with the described methods in the previous section.

PVA/IND samples were mixed with 50 mL phosphate buffer pH 7.2 according to USP and stirred up (450 rpm) for 10 min at 45 °C. Subsequently, the samples were cooled down at room temperature, filled up with Acetonitrile, and stirred up again for 15 min.

PVA/KTZ samples were placed in the volumetric flask, mixed with 50 mL 0.1 M HCL at room temperature, and stirred up at 450 rpm until everything dissolved. Then, the volumetric flask was filled up with Methanol and stirred up for 15 min.

#### 2.2.16. HPLC Measurements

HPLC tests were carried out using the HPLC 1260 Infinity II (Agilent Technologies Cooperation, Santa Clara, CA, USA) to analyze the content and the drug concentration of KTZ and IND in the tablets.

PVA/KTZ 3DP tablets were analyzed at a wavelength of 225 nm using HPLC, which was equipped with a Waters µBondapak RP18e 300 mm column featuring a particle size of 10 µm. The mobile phase consisted of two eluents in a ratio of 70:30 (Eluent 1: Eluent 2). Eluent 1 was a mixture of diisopropylamine/methanol (2 mL/5 L), while Eluent 2 comprised an ammonium acetate solution at a concentration of 5 g/L (*w*/*w*%). The system operated at an isocratic flow rate of 2 mL/min, with the column temperature maintained at 40 °C. The autosampler was programmed to inject 5 µL of sample every 8 min. The method was developed internally and optimized to ensure effective peak and area separation between the excipient and the API. A five-point calibration was conducted to confirm linearity within the measurement range. The results were validated by including a control standard in each measurement series.

PVA/IND tablets were analyzed at a wavelength of 254 nm. The separation was executed with a Waters µBondapak RP18e 300 mm column with a particle size of 10 µm. The mobile phase was a mixture of acetonitrile and buffer solution for IND 1 L/1 L. The isocratic flow rate was set to 1 mL/min and the column temperature at 20 °C. The injection volume amounted to 15 µL every 7 min. The method is derived from the USP and was conducted using a 5-point calibration. The results were verified with a control standard in each measurement series.

#### 2.2.17. Dissolution

The dissolution tests were performed using the Sotax AT7 smart (Sotax, Lörrach, Germany) dissolution tester following the USP apparatus 2 method.

For the analysis of PVA/IND tablets were analyzed in 900 mL simulated gastric fluid (SGF) at 37.5 °C ± 0.5 °C with a paddle speed of 75 rpm. The online UV-VIS method was performed at 318 nm. Samples were taken at 5, 10, 15, 20, 25, 30, 45, 60, 75, 90, 105, 120, 150, 180, 240, 300, and 360 min.

PVA/KTZ tablets were analyzed with an offline UV-VIS method at a paddle speed of 75 rpm in 900 mL 0.1 M HCL at 37.5 °C ± 0.5 °C. Samples were obtained and filled into HPLC vials and analyzed accordingly with the HPLC method. For solubility enhancement, KTZ tablets were dissolved in 100 mL in fasted state simulated intestinal fluid (FaSSiF) (pH = 6.5) The dissolution tests were conducted in Erlenmeyer flasks which were placed on a shaking platform (TiMix 5, Edmund Bühler GmbH, Bodelshausen, Germany) inside an incubator (TH 15, Edmund Bühler GmbH) shaking at 450 rpm at a temperature of 37.5 °C. Samples were taken at 5, 15, 30, 60, 90, 120, 240, and 360 min. The method was specifically adjusted to observe the supersaturation of dissolved KTZ, particularly from the tablets with higher infill volumes.

## 3. Results

### 3.1. Analysis of Particle Size Distribution and Flow Properties of Milling Fractions

The printer is designed for the milling of pellets ranging from 2 to 4 mm in size. To evaluate the use of powders and to identify potential limits in particle size, both commercial PVA and ground PVA flakes in various milled fractions were analyzed. It is noted that at a particle size limit of 42.68 µm, 50 percent of the commercial PVA 4-88 volume is included. At D90, the maximum particle size is 101.75 µm. These values indicate that 50 percent of the volume comprises very small particles. A broad spectrum of particle sizes was generated by varying the sizes of the sieve inserts, allowing for an evaluation of processability. For fraction 1, a sieve insert with a mesh size of 500 µm was used, for fraction 2 a 750 µm sieve insert, for fraction 3, a 1000 µm sieve insert, and for fraction 4, a 2000 µm sieve insert was employed. The produced fractions had a narrow range. The results of the measured particle sizes are listed in Table 3.

To determine the flow properties of the various sieve fractions, the bulk and tapped densities were measured, as well as the angle of repose. Additionally, the Hausner ratio and the Carr index were calculated. The results can be found in Table 4. From these results, it is evident that fractions 4 and 3 exhibit acceptable Hausner Factors and Carr Indices according to USP evaluation criteria. However, both values for fraction 2 were in the poor range. The angle of repose for fractions 4, 3, and 2 was fair.

### 3.2. Rheology

**Figure 2:**
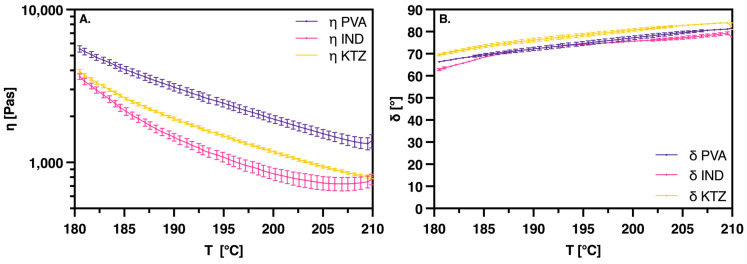
(**A**): Complex viscosity against temperature. (**B**): Phase angle δ against temperature; for PVA and PVA/API mixtures, API proportion is 10%, means of n = 3 ± SD.

Rheological investigations were conducted on both pure PVA and physical mixtures of PVA with KTZ or IND. These studies revealed a decrease in viscosity across all systems with an increase in temperature (Figure 2A). Both APIs significantly influenced the viscosity of PVA, markedly reducing the melt viscosity. Among them, IND had the most pronounced effect, especially at lower temperatures. The greatest disparity in viscosity was observed around 180 °C, where the mixture containing IND exhibited a viscosity approximately 2000 Pas lower than that of pure PVA, while the mixture with KTZ showed a reduction of about 1600 Pas.

To further compare the viscoelastic properties of the API mixtures with those of pure PVA, the phase angle δ was analyzed (Figure 2B). This angle, which is measured between the applied stress and the resulting strain in an oscillatory test, serves as a direct indicator of the material’s balance between elastic and viscous behavior [29]. A phase angle greater than 45° signifies a dominantly viscous material, whereas an angle less than 45° indicates a predominance of elastic behavior. The addition of the APIs did not markedly alter the viscoelastic behavior of PVA, with the samples exhibiting predominantly viscous behavior throughout the entire temperature range of 180 °C to 210 °C. Notably, the mixture with KTZ demonstrated a slightly higher phase angle δ, indicating a more pronounced viscous behavior compared to the mixture with IND and pure PVA.

### 3.3. Determination of FF and Printing Settings for the AMDD Process

With the Freeformer, it was possible to process fraction 2 (sieve size: 750 µm). At smaller particle sizes, processing was no longer feasible. Therefore, the 750 µm fraction was selected for further investigations with API and processing with the Freeformer.

**Figure 3:**
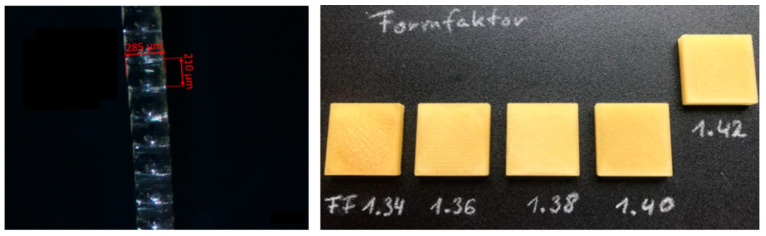
(**Left**): Strand produced with HME pellets containing PVA 4-88 and 10% KTZ; (**Right**): Determination of FF using five cubes with different FF (20 mm × 20 mm × 4 mm).

A difference between the FDM process and the AMDD process is the determination of the FF (Form Factor) to enable printing with new materials. The form factor, integrated into the slicer software, defines the space allocated for the deposited droplets. Hence, the droplet geometry initially guides the selection of a preliminary form factor for a cube. The FF should be set to a low value to avoid overfilling the object. The droplet geometry is influenced by the material, the volume-to-droplet ratio, and the temperature, which affects viscosity. At higher temperatures and thus lower viscosities, droplets tend to elongate. Figure 3 exemplarily shows a strand produced with HME granulate containing PVA and 10% KTZ. Taking the produced strand with a width of 285 µm and a height of 210 µm as an example, a preliminary form factor of 1.35 is derived. Following the printing of the five cubes and a visual and tactile examination, an FF of 1.33 was determined and used for subsequent printing processes for this material. Figure 3 exemplifies the determination of the FF for the wet-granulated placebo intermediate. Here, after examination, an FF of 1.36 was selected.

The temperatures for the printing process for each intermediate were determined in reference to the thermal values obtained from the extrusion process. These results demonstrate that the printing of the strand and the cubes with the intermediates was successful, thus enabling the production of 3DP tablets.

### 3.4. Tablet Characterization

#### SEM Images

The production of 3DP tablets from all intermediates, incorporating both active ingredients and placebo, was successful. This included the successful creation of 3DP tablets with various infill volumes as well as tablets with 100% infill.

**Figure 4:**
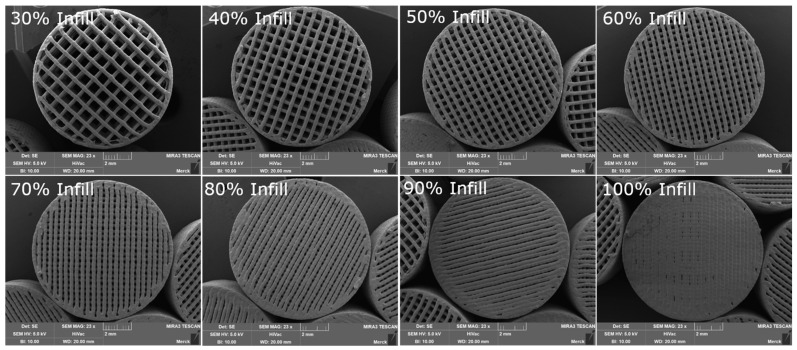
SEM images of 3DP tablets created with HME pellets (variation of infill volume).

**Figure 5:**
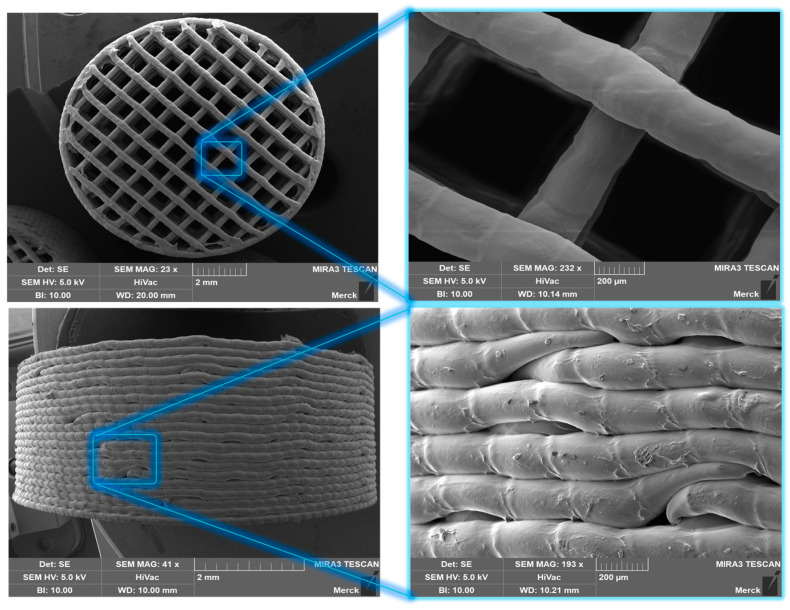
SEM image of a 3DP tablet with 30% infill volume produced with HME pellets.

Figure 4 displays the different infill volume placebo tablets made from the HME intermediate. It can be observed from the figure that as the infill volume increases, the distance between individual strands decreases until, at 100% infill volume, they adhere to each other and form an almost completely closed surface. Additionally, Figure 5 illustrates the individual layers of the tablet and the boundaries of droplets within the droplet strand.

### 3.5. Mass Distribution

**Figure 6:**
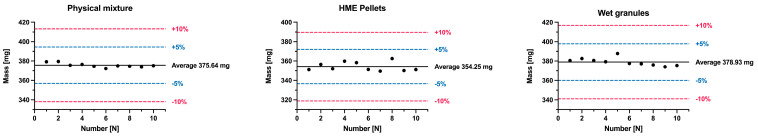
Mass distribution of 3DP placebo tablets produced with different intermediates n = 10 (specifications according to Ph. Eur.).

To ensure a homogeneous printing process with various intermediates, the mass distribution of the 3DP tablets was examined. Figure 6 illustrates the individual tablet masses printed from various placebo intermediates. It is noticeable that the masses deviate only slightly from the calculated average. The masses of the printed tablets exhibit only very slight variations, which remain below the threshold of ±5% for all intermediates. Therefore, the printing process is very homogeneous and meets the criteria of the Ph. Eur., confirming that the mass consistency is not influenced by the different intermediates.

**Figure 7:**
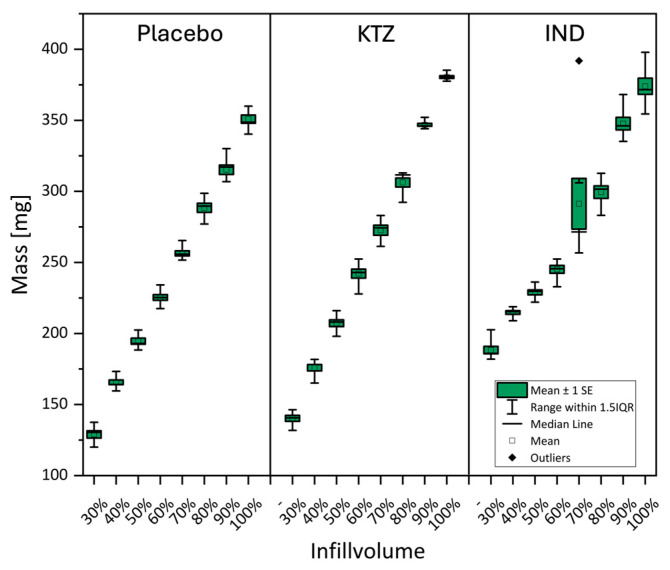
Mass deviation of 3DP tablets produced with hme pellets and APIs (10%).

The influence of API and various infill volumes on the mass was then assessed using 3DP tablets made from HME pellets. In Figure 7, the results of the mass versus infill volume are presented in a candlestick chart, revealing significant differences. It is observed that KTZ tablets do not exhibit significant deviations or outliers. For IND, a notable outlier is observed at 70% infill volume, where the standard deviation is also high. These values indicate that IND does not allow for reliable printing. This mass deviation, particularly from an infill volume of 70%, was anticipated during the tablet printing, as the material appeared slightly foamed.

### 3.6. PXRD Analysis

**Figure 8:**
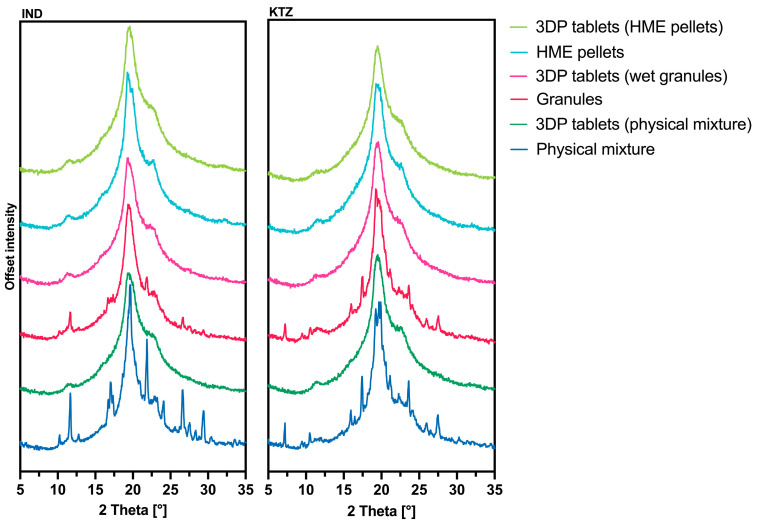
PXRD patterns of the various intermediates and the printed tablets printed from them, (**left**): PVA with 10% IND, (**right**): PVA with 10% KTZ.

To determine whether the selected model compounds could be processed into ASDs through the AMDD procedure, a PXRD analysis was conducted. This analysis was performed on the physical mixture, the wet granulates, the HME pellets, and the tablets printed from them. The aim was to investigate whether the double melting process in the production of HME pellets offers advantages in the manufacturing of printed ASDs compared to the two other starting materials. The findings are presented in Figure 8.

The distinctive peaks of IND in the diffraction pattern occur at 11.68 °2Theta and 21.86 °2Theta. These peaks are evident in the intermediates of both the physical mixture and the wet granulation process. However, the HME pellets and the 3DP tablets from all intermediates exhibit an amorphous structure.

For KTZ, characteristic peaks are identified at 17.36 °2Theta and 23.60 °2Theta. In a similar pattern, all intermediates appear amorphous after 3DP. The diffraction patterns of the 3DP tablets show no crystalline peaks, indicating their amorphous nature.

### 3.7. DSC Analysis

**Figure 9:**
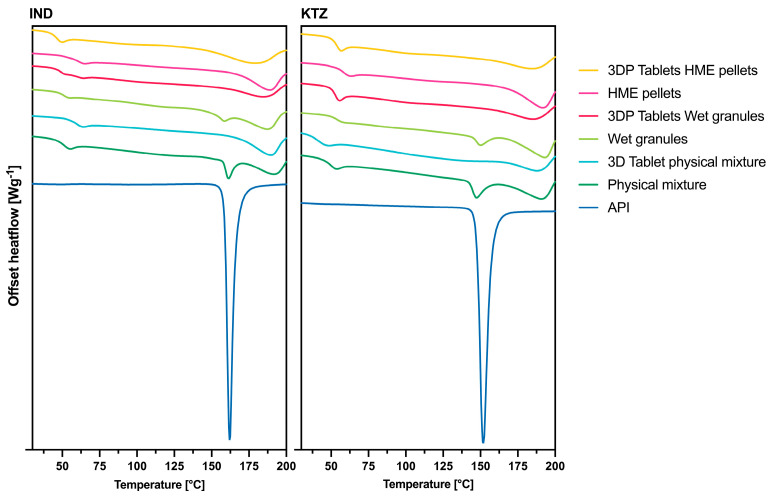
DSC thermograms of the crystalline API, the various intermediates (API-PVA combinations with a drug load of 10%), and the tablets printed from these intermediates, also with a drug load of 10%. (**Left**): PVA with IND; (**Right**): PVA with KTZ.

To further explore the amorphization of the 3DP tablets, a comprehensive DSC analysis was conducted. The outcomes of these analyses are detailed in Figure 9.

IND exhibited a melting point at 161 °C, a characteristic discernible in both the physical mixture and wet granulation. No crystalline forms were detected within the HME pellets, or the 3DP tablets from all intermediates, indicating that the drug underwent complete amorphization during the printing process.

Similarly, KTZ displayed a melting point at 150 °C. This melting event was present in both the physical mixture and wet granulation. In the case of HME pellets, the melting point of the drug was absent. The 3DP tablets further confirmed that KTZ became amorphous during the printing process. Additionally, the crystalline regions of PVA, typically ranging between 170 °C to 200 °C, were notably reduced in intensity compared to the intermediates.

### 3.8. Content Analysis with NMR and HPLC

The drug content was determined by HPLC and NMR. Table 5 summarizes the drug content of the API formulations before and after the 3DP process. The targeted loading of each formulation was 10% API. The determined HPLC and NMR contents are in good agreement for all samples except for the physical mixtures. The bigger deviation for the latter is most likely due to a pronounced inhomogeneity of the sample itself. All NMR-based contents show a slightly higher value than the HPLC-based contents. This is also true for the pure API starting materials. Hence, this slight bias can be assigned to the method itself. KTZ formulations show a good match to the targeted 10% drug load throughout all formulations, both before and after the 3DP process. From this, it can be concluded that the API is stable and can be processed even in the presence of the thermal and mechanical stress of the 3DP process. In contrast to this, the IND formulation shows a systematic decrease in the API content after the 3DP process. This finding indicated a decomposition of IND during the 3DP process.

**Figure 10:**
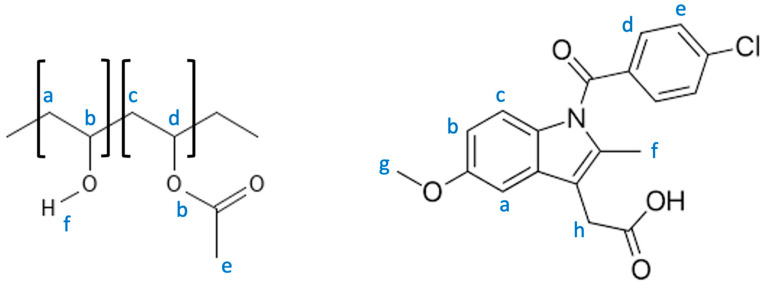
Molecular structure of PVA (**left**) and the model compound IND (**right**).

**Figure 11:**
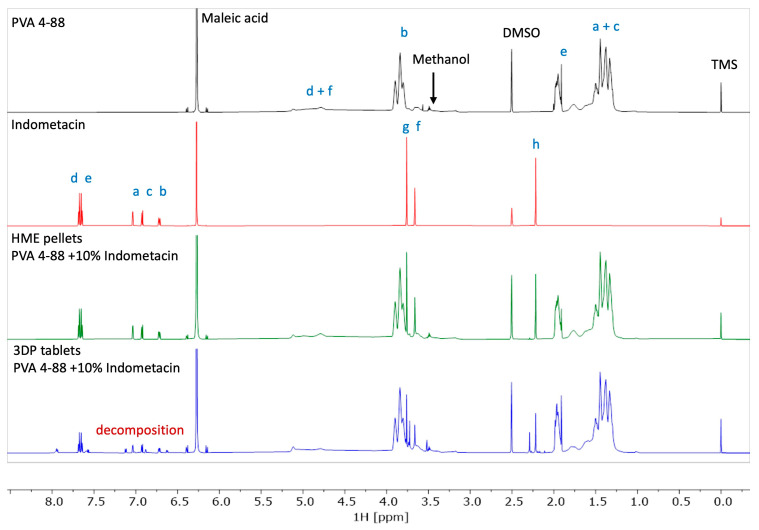
NMR results of PVA, IND, HME pellets IND (10%), and 3DP tablets of HME pellets.

**Figure 12:**
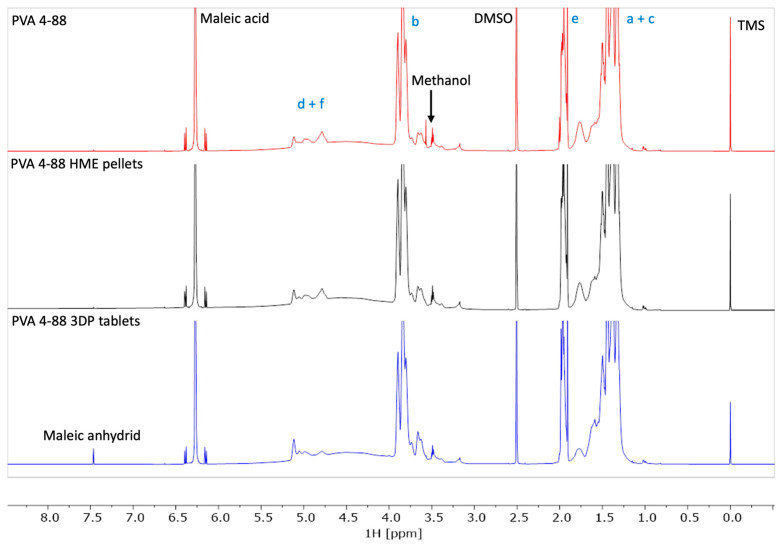
NMR results of pure PVA, HME pellets, and 3DP tablets of HME pellets.

In Figure 10, the structural formular of IND and PVA together with the assignment used in Figure 11 and Figure 12, are depicted. Figure 11 shows the NMR stacked plot of pure PVA (black) and pure IND (red), as well as the processed HME pellets (green) and the 3DP tablet (blue). Since the NMR spectrum of a compound is very characteristic of its structure, an eventual decomposition is typically indicated by the presence of additional signals or a shift in the NMR line positions. As can be clearly seen, the 3DP printed tablets show additional signals mainly in the aromatic region of IND, thereby confirming and proving the decomposition of IND in the printing process.

The stability of the PVA polymer itself was also tested and is depicted as a stacked plot of the starting material and the HME and 3DP printed processed material in Figure 12. All spectra show an identical 1H NMR pattern for the polymer. There are no indications of decomposition. The starting material contains a low level of methanol, which is no longer detected in the processed materials. The lower spectrum shows an additional signal originating from maleic acid anhydride. This is formed in situ in DMSO solutions due to a slightly prolonged storage of about 2h prior to the NMR spectra measurement.

### 3.9. In Vitro Dissolution Studies

**Figure 13:**
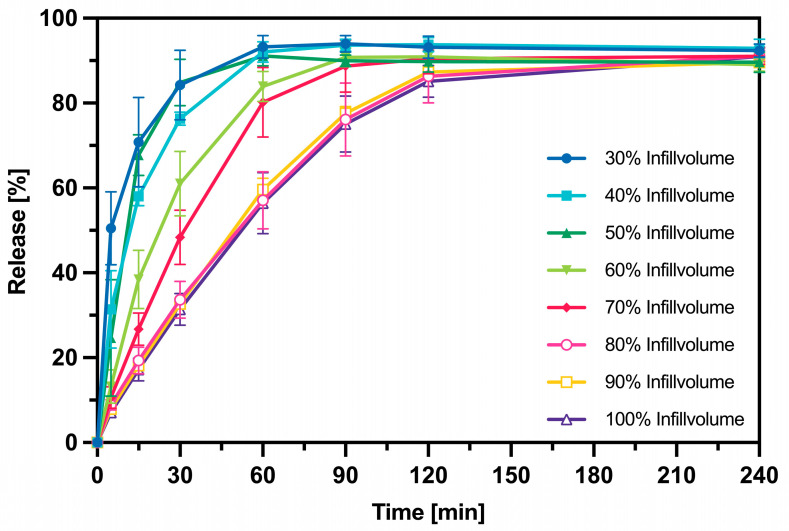
Left: Dissolution profile of 3DP tablets with different infill volumes in 900 mL 0.1 M HCL, Mean value ± SD, n = 3.

Since the previous analyses showed no differences between the intermediates in terms of print quality and the solid-state character of the ASDs, the release and solubility enhancement experiments were conducted only with one intermediate, the HME pellets. Figure 13 shows the results of the release performance of 3DP tablets containing KTZ (*w*/*w* 10%). The release kinetics of the tablets with 100%, 90%, and 80% infill volumes are very similar, releasing the API slowly, reaching the 80% release mark only after 120 min. As the infill volume decreases, the release rate increases, and with 30% infill, tablets were produced that exceeded the 80% release mark within just 30 min.

**Figure 14:**
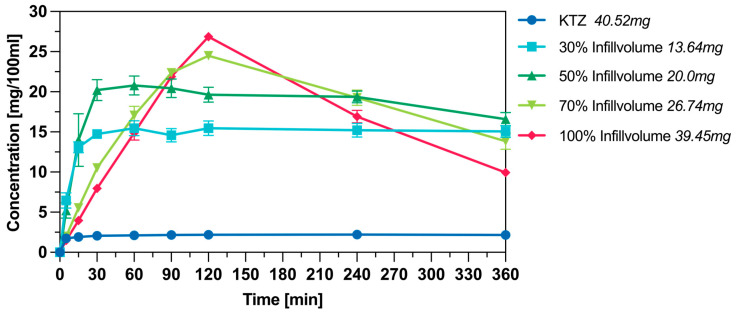
Dissolution profile of 3DP tablets with 10% KTZ in 100 mL FaSSiF (pH = 6.5), Mean Value ± SD, n = 3.

For release investigations in FaSSiF, 3DP tablets with 30%, 50%, 70%, and 100% infill volume were used. With all tested infill volumes, the concentration of dissolved API was markedly higher compared to the pure crystalline form. For the crystalline KTZ, only about 5% dissolved over the course of the measurement period. The tablets with 30% and 50% infill volume reached their targeted concentrations. Whereas the tablets with 70% and 100% infill volume reveal a supersaturation behavior. For the tablets with 70% infill, a maximum concentration was observed at 120 min, reaching 92% of the API used. A similar trend was noted for tablets with 100% infill volume, where the maximum concentration reached 68% of the API in solution. The results can be seen in Figure 14.

**Figure 15:**
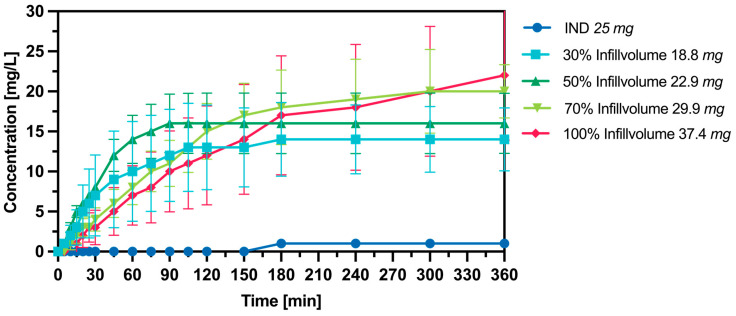
Dissolution profile of 3DP tablets produced from HME pellets with different infill volumes in 900 mL SGF Mean Value ± SD, n = 3.

The results of the release performance of IND 3DP tablets in SGF are presented in Figure 15. The shows the amount of drug in the tablet. In the experiments with IND, higher concentrations of IND in solution were observed for all infill variations of the 3D-printed tablets compared to the crystalline IND. However, the aim concentration is not achieved by the printed Tablets. This could be again the decomposition of the IND tablets discussed in the chapter (drug content). The observed concentrations of the release study coincide with the drug content, which was determined with NMR and HPLC.

## 4. Discussion

The literature generally describes that the flow properties of the starting materials are crucial for their processing using extrusion-based 3DP methods, as they are responsible for the material flow and the feeding from the hopper into the extruder screws [30,31]. For the Freeformer 3D printing system, the particle size distribution appears to have a greater influence. Despite the recommended particle size of 2–4 mm, processing with an average particle size of 200 µm was achieved, thus pushing the limits of the AMDD System in terms of particle size. A fraction with an average particle size of 150 µm particle size could not be processed, which is attributable to inadequate material conveyance by the screw. The screw’s simple structure, containing only a basic thread, does not favor material conveyance. A screw design incorporating conveying and kneading elements, like an extruder, could resolve this issue. M. Saviano et al. demonstrated in a study on the FDM process that particle size significantly affects the miscibility between the polymer and APIs. Fine particles performed the best in this context. The release profiles were almost superimposable, and the API distribution was very homogeneous at a particle size between 250–600 μm, with a variation of 9.55% ± 0.46% [32]. The Freeformer system can meet these requirements for particle size, thus enabling the direct printing of powder mixtures.

For the printing of API–polymer mixtures using melt-based 3DP processes, the melt viscosity is crucial. It is known that various APIs have a plasticizing effect on some polymers. The reduction in viscosity occurs because the API molecules enter between the long polymer chains, thus reducing the intermolecular forces between the polymer chains [33,34]. This often coincides with a decrease in the Tg and/or the Tm. This effect is very likely applicable to KTZ and IND in combination with PVA as well. The reason why IND exhibits a stronger plasticizing effect could be due to its lower molecular weight, allowing more individual IND molecules to insert themselves between the polymer chains for the same mass proportions of API. Through rheological investigations, we hoped to gain a better understanding of the different form factors measured during the evaluation of printing parameters. These correlate with the reduction in viscosity of the polymer melt: the lower the viscosity, the smaller the form factor. The viscoelastic properties provide an indication of the polymer’s relaxation time. In highly elastic systems with a dominant storage modulus, i.e., a phase angle δ under 45°, the polymer melt can expand again after compression through the nozzle. In extrusion, this is referred to as “Die Swell” [33]. Here, the two APIs seem to have only a neglectable influence on the fundamental viscoelastic behavior of PVA; the systems remain predominantly viscous. Within the measured range, which covers the 3D printing temperature ranges, both active ingredients appear to have only a negligible effect on the fundamental viscoelastic behavior of PVA; the systems remain predominantly viscous. The viscoelastic behavior in polymer melts is typically temperature-dependent, and changes in viscoelastic properties often indicate a thermal event, such as a melting point or glass transition temperature (Tg). The APIs used, IND and KTZ, only slightly shift the melting point to a lower temperature, which remains outside the printing temperature range. Therefore, it is assumed that relaxation in the case of PVA or PVA-API mixtures has a lesser impact on the form factor. The unique aspect of AMDD technology, the individual form factor determined beforehand for the formulation, offers a great advantage in printing the API–polymer combinations. In traditional FDM printing processes, responses to viscosity changes in the polymer melt due to APIs, such as adjustments to temperature or print speed, are made [35]. With the AMDD printing system, the printing parameters could remain unchanged for the KTZ mixture compared to the placebo printing. This could offer an advantage in applications with varying drug load requirements, for example, in dose finding in clinical studies or in personalized medicine. Thus, minor API-induced viscosity fluctuations could be compensated by the form factor, allowing printing with the same parameters, which facilitates the comparability of the product.

For the successful production of amorphous HME pellets with IND and KTZ, extrusion temperatures of at least 200 °C were chosen, which are above the melting points of KTZ (Tm = 151 °C [22,36]) and IND (Tm = 160 °C [37]). Additionally, all manufactured 3DP tablets from all intermediates were in an amorphous state. Due to the previous amorphization of the pellets by HME, the amorphous state of the 3DP tablets was expected. The amorphization of the wet granulate and the physical mixture occurred through the AMDD technology which can convert crystalline starting material into an amorphous form. These findings were confirmed by DSC and PXRD analysis of the 3DP tablets. The absence of an additional melting point or an extra glass transition point in the thermograms, beyond that of pure PVA, indicates that the API is present in an amorphous form within the polymer [38].

When examining the solid-state status of APIs, it is essential to consider their polymorphism. KTZ is straightforward in this regard, as no polymorphs of KTZ are known [39]. IND, on the other hand, exists in several polymorphic forms, which can be categorized into the stable γ-form, the less stable α-form, and the metastable forms β, δ, ε, η, ζ, and τ [40]. The formation of a particular polymorph can be influenced by the manufacturing process. Examples of polymorph production include crystallization from melts or from solutions, with the choice of solvent potentially impacting which polymorph is formed. In our case, the starting material IND was in the stable γ-form [40]. Following 3D printing, the API was found to be amorphous within the polymer matrix. We did not observe any polymorph formation. Furthermore, the granulation step did not lead to any change in configuration.

The production of 3DP tablets from all intermediates with 10% KTZ and as a placebo resulted in homogenous and uniform tablets. This is a clear indication of the AMDDs printing precision. The homogeneous material discharge can be attributed to the successful determination of the fill factor. Thus, accurate and consistent masses as well as drug contents were achieved.

The production of 3DP tablets from HME pellets made of PVA and 10% IND as starting material resulted in inhomogeneous tablets in terms of mass and appearance, with low drug content and release.

The decomposition of IND is likely due to the elevated working temperatures during processing. Since no decomposition occurred during melt extrusion, it is probable that the extended residence time of the API in the heating chamber of the 3D printing system plays a crucial role; under our settings, this residence time is approximately 6 min. Another factor potentially contributing to the decomposition, in addition to the prolonged residence time, may be the high shear forces generated by the AMDD printer. This system operates at pressures exceeding 400 bar. Literature suggests that both increased screw speeds and mechanical shear forces, created by resistance and pressure, can lead to drug decomposition [41,42,43]. Moreover, the production of HME pellets using an extruder was successful, as it operates at pressures below 100 bar, while the production of 3DP tablets directly from the physical mixture and wet granulate with the AMDD printer led to the decomposition of the drug.

When examining the results of the in vitro release study of the 3DP tablets made from HME pellets with 10% KTZ, one observes a control of the release kinetics through the infill volume. As the infill volume increases, the tablets release the drug more slowly. These results were also achieved in the study by Goyanes et al. [17]. Beyond an infill volume of 80%, the tablets were released at the same rate. This could be related to the distance between the strands, which are close to each other at an infill volume of 80% and above. During the release, the tablet swells, forming a gel layer that causes a delayed drug release. With infill volumes below 80%, the strands are farther apart, allowing for a quicker release. All 3DP tablets with different infill volumes were able to release completely due to the good solubility of KTZ in 0.1M HCl, as seen in the study by Ullrich et al. [44]. Additionally, KTZ is a weak base and dissolves well in solutions with a pH of 1 [45]. The 3D-printed tablets made from HME pellets, in which KTZ was present in an amorphous form, improved the solubility of KTZ in FaSSIF (pH = 6.5) compared to crystalline KTZ. This result was anticipated since the amorphization of the active substance occurred after the 3DP process. Gottschalk et al. were also able to produce 3DP tablets using FDM (Fused Deposition Modeling) and powder jet binding, which increased the solubility of KTZ [14]. Furthermore, Roche et al. in their review article present various studies that demonstrate the enhancement of solubility for BCS (Biopharmaceutics Classification System) Class II and IV drugs using FDM [46]. ASDs have the potential to both increase the solubility of the drug and improve the stability of the API compared to crystalline drugs [47]. Of course, the stability and storage of the ASDs must also be considered and analyzed to counteract the crystallization of the API in the matrix.

## 5. Conclusions

The AMDD 3D printing technology was successfully utilized to print uniform and homogenous tablets from pure PVA as well as with PVA and 10% KTZ. The high accuracy of dosing, achieved by depositing individual defined droplets, is reflected in a good uniformity of mass. Moreover, it was demonstrated that the release kinetics of the 3D printed tablets, using PVA as the polymer matrix, could be controlled and modified by altering the infill volumes.

Furthermore, it has been demonstrated that the AMDD system is capable of independently generating amorphous solid dispersions without requiring any additional preliminary extrusion steps. Consequently, the solubility of poorly soluble pharmaceuticals can be enhanced using the AMDD method.

The experiments indicated that the AMDD system can accommodate various intermediates. In addition to the manufacturer-recommended starting material, such as pellets, granules, and powders were also directly processed. With the currently employed single-screw extruder system, the limit for particle size is approximately 200 µm. However, with modifications to the feeding mechanism, such as equipping it with a powder feeder and dosing screws, it is highly likely that smaller particles could also be processed. This is because, based on the experiments conducted, the main limiting factor was ensuring the consistent feeding of material into the extruder.

## Figures and Tables

**Table 1:** HME process parameters for PVA and PVA/API extrudates.

	Pure PVA	10% IND + 90% PVA	10% KTZ + 90% PVA
Nozzle [°C]	210	200	200
Zone 3–7 [°C]	210	200	200
Zone 2 [°C]	150	120	120
Zone 1 [°C]	80	76	76
Feeding rate [kg/h]	0.2	0.5	0.45
Die pressure [bar]	6–7	16–28	18–20
Melt temperature [°C]	205	193	194
Torque [%]	41	59	50
Screw speed [rpm]	300	400	450
Speed conveyor belt [m/min]	1.45	3.91	4.52

**Table 2:** 3D printing parameters.

	Pure PVA			10% IND + 90% PVA			10% KTZ+ 90% PVA		
Material	HME Pellets	Physical Mixture	Granules	HME Pellets	Physical Mixture	Granules	HME Pellets	Physical Mixture	Granules
Temperature nozzle [°C]	210	210	210	200	200	200	210	210	210
Temperature Zone 2 [°C]	190	190	190	180	190	190	190	190	190
Temperature Zone 1 [°C]	170	170	170	160	160	170	170	170	170
Temperature Feeding area [°C]	45	45	45	45	45	45	45	45	45
Chamber [°C]	80	80	80	80	80	80	80	80	80
Discharge [%]	70	70	70	70	70	70	70	70	70
Form factor	1.36	1.36	1.36	1.20	1.28	1.38	1.33	1.33	1.33

**Table 3:** Particle sizes at d10, d50, and d90 for commercial PVA and the milling fractions.

	Commercial PVA 4-88	Fraction 1	Fraction 2	Fraction 3	Fraction 4
D10 [µm]	12.02	36.43	47.45	51.33	116.90
D50 [µm]	42.68	149.40	198.35	221.51	441.99
D90 [µm]	101.75	350.87	473.47	534.03	1013.5
Span	2.102	2.105	2.148	2.179	2.029

**Table 4:** Flow properties of commercial PVA and the milling fractions.

	Bulk Density [g/mL]	Tapped Density [g/mL]	Hausner Factor	Carr-Index	Angle of Repose [°]
Commercial PVA 4-88	0.54	0.75	1.4	27	32.5
Fraction 1	0.54	0.72	1.3	25	36.3
Fraction 2	0.45	0.63	1.4	28	36.1
Fraction 3	0.49	0.66	1.3	25	35.8
Fraction 4	0.48	0.64	1.3	25	36.6

**Table 5:** Drug content of the formulations derived from various intermediates (HME pellets, granulates, and physical mixtures) before and after the 3D printing process, determined by NMR and HPLC analyses.

Material	Drug (10% Loading)	NMR Measurement 1 [%]	NMR Measurement 2 [%]	Assay [%] (HPLC)
HME-Pellets	IND	9.8	10	9.6
3DP tablets (HME pellets)	IND	6.1	6.3	4.7
Granules	IND	11.7	11.4	10
3DP tablets (granules)	IND	7.7	7.4	5.3
Physical mixture	IND	13.2	11.8	8.8
3DP tablets (physical mixture)	IND	4.7	4.9	3.5
HME-Pellets	KTZ	9.8	9.9	9.1
3DP tablets (HME-pellets)	KTZ	9.5	9.6	8.0
Granules	KTZ	9.8	9.4	8.8
3DP tablets (granules)	KTZ	9.6	9.4	7.9
Physical mixture	KTZ	6.5	6.6	8.7
3DP tablets (physical mixture)	KTZ	8.5	8.5	7.7

## Data Availability

The data presented in this study are available in this article.

## Supplementary Materials

The following supporting information can be downloaded at: https://www.mdpi.com/article/10.3390/pharmaceutics16121501/s1, Figure S1: Screw configuration. Image created with the ScrewConfig software (Version 1.42(2) Thermo Fisher Scientific Inc., Waltham, MA, USA).
