# Supplementary figures and images for: Exploring 3D Printing in Drug Development: Assessing the Potential of Advanced Melt Drop Deposition Technology for Solubility Enhancement by Creation of Amorphous Solid Dispersions
Source: Pharmaceutics. 2024 Nov 22;16(12):1501. doi: 10.3390/pharmaceutics16121501 (PMC11679577; doi:10.3390/pharmaceutics16121501)

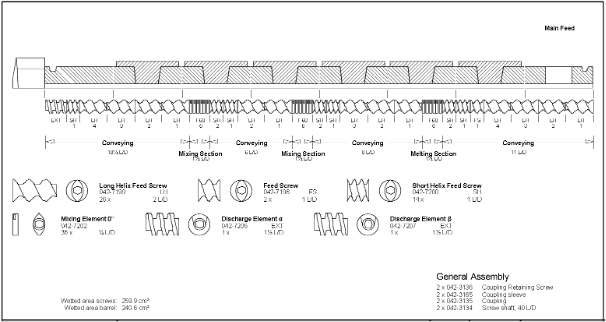

Supplement: Supplementary file 1 [file pharmaceutics-16-01501-s001.zip › pharmaceutics-3288124-Figure S1.png]
